# CUTANEOUS INVOLVEMENT IN ANGIOIMMUNOBLASTIC T-CELL LYMPHOMA

**DOI:** 10.4103/0019-5154.70704

**Published:** 2010

**Authors:** Evangelia Papadavid, Ioannis Panayiotides, Marianna Dalamaga, Alexandros Katoulis, Theofanis Economopoulos, Nikolaos Stavrianeas

**Affiliations:** *From the Department of Dermatology, Internal Medicine & Pathology, University of Athens, ATIIKON General Hospital, Athens, Greece*

**Keywords:** *Angioimmunoblastic T-cell lymphoma*, *cutaneous involvement*

## Abstract

Angioimmunoblastic T-cell lymphoma (AITL) is an aggressive non-Hodgkin’s nodal peripheral T-cell lymphoma characterized by general lymphadenopathy, night sweats, fever, hepatosplenomegaly, polyclonal hypergammaglobulinemia, and cutaneous involvement. We present a rare case of AITL cutaneous involvement mimicking toxic erythema recurring with AITL relapse and suggesting a precursor of disease progression.

## Introduction

The cutaneous findings of angioimmunoblastic T-cell lymphoma (AITL) most commonly consist of a maculopapular eruption on the trunk mimicking toxic erythema. The rash is persistent and usually misdiagnosed as toxic erythema due to drug eruption or viral exanthem.[1] A skin biopsy reveals histological and immunohistochemical findings and leads to the diagnosis of cutaneous AITL. In doubtful cases, molecular studies of clonal TCR-γ-chain gene rearrangement in the skin and lymph nodes are helpful.[2,3]

## Case Report

**Figure 1:**
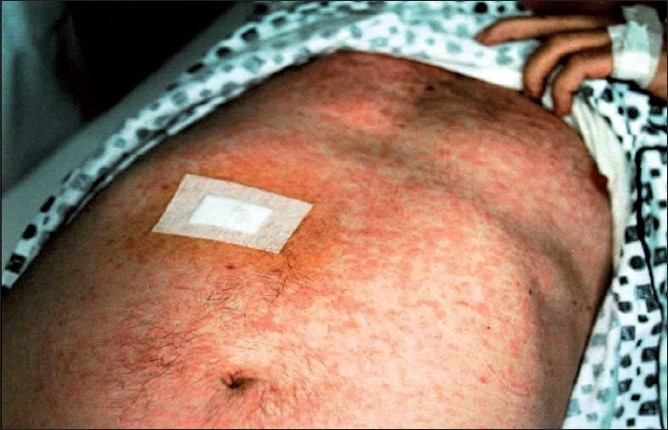
Widespread macular erythema on the trunk

**Figure 2:**
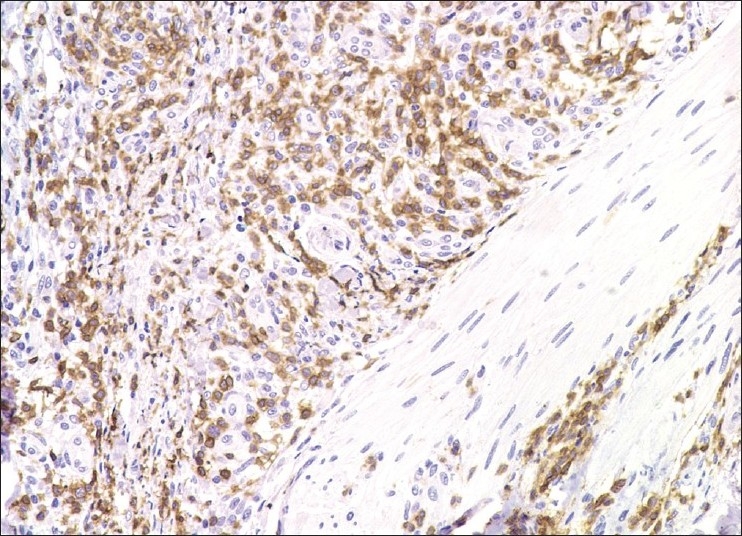
A dense perivascular cellular infiltration consisting of mature lymphocytes, histiocytes, a small number of eosinophilic polymorphonuclear leucocytes and few small to medium size lymphoid cells with oval or cleaved nucleus positive for CD3 was seen in the dermis (CD3, × 20)

A 61-year-old man with a 2-year history of AITL presented to the Dermatology Department with a widespread macular erythema on his trunk and extremities mimicking toxic erythema [Figure 1]. The rash, first appeared at the time of diagnosis, was persistent and misdiagnosed as toxic erythema due to drug eruption and disappeared only with complete remission after chemotherapy COP. After two years, the rash relapsed with AITL progression. His virology tests for recent EBV, CMV, HSV1, HSV2, HZV, HTLV-1, and HTLV-2 infections were negative. A skin biopsy performed at disease relapse was diagnostic of skin involvement in AITL. The epidermis did not show any specific changes, but a dense perivascular cellular infiltration consisting of mature lymphocytes, histiocytes, a small number of eosinophilic polymorphonuclear leucocytes, and few small to medium size lymphoid cells with oval or cleaved nucleus was seen in the dermis. The majority of the cells were positive for CD3 [Figure 2] and negative for CD79a, CD68/PGM1, CD30, CD4, and CD8. The patient’s condition progressed despite chemotherapy and had a fatal course few months after the second relapse.

## Discussion

AITL is an aggressive non-Hodgkins nodal peripheral T-cell lymphoma characterized by general lymphadenopathy, night sweats, fever, hepatosplenomegaly, polyclonal hypergammaglobulinemia and cutaneous involvement. The cutaneous findings most commonly consist of a maculopapular eruption on the trunk. However, purpura, infiltrated or urticarial plaques, papulovesicular lesions, nodules, and erythroderma have also been reported.[1] Forty-four percent of patients with AITL experience a nonspecific maculopapular dermatitis, which precedes other clinical symptoms by at least several weeks[1] suggesting that AITL should be included in the differential diagnosis of any maculopapular eruption of unknown etiology accompanied by lymphadenopathy. Histologic findings in the lymph node are characteristic, while those in the skin may be very subtle, showing only mild lymphoid infiltrate. In our case, the clinical history of AITL in addition to the clinical, histological, and immunohistochemical findings led to the diagnosis of cutaneous AITL. In doubtful cases, molecular studies of clonal TCR-γ-chain gene rearrangement in the skin and lymph nodes are helpful.[2,3] Although its pathogenesis is not clear, AITL is a peripheral T-cell lymphoma of mature T-cells secreting several cytokines, which may help in predicting the biological behaviour of such an aggressive disease.[4]

We present a rare case of AITL cutaneous involvement mimicking toxic erythema recurring with AITL relapse and suggesting a precursor of disease progression. Given the allergy of the patient to the most frequently administered antibiotics, the risk of misdiagnosing the origin of such an eruption is present. In most of the cases, awareness of the history of AITL with ancillary studies, including clonality testing for T-cell receptor gene rearrangement, is crucial for reaching an accurate diagnosis.[3,4]
